# Interplay between MexAB-OprM and MexEF-OprN in clinical isolates of *Pseudomonas aeruginosa*

**DOI:** 10.1038/s41598-018-34694-z

**Published:** 2018-11-07

**Authors:** Gertrudis Horna, María López, Humberto Guerra, Yolanda Saénz, Joaquim Ruiz

**Affiliations:** 1 Barcelona Institute for Global Health, ISGlobal, Hospital Clínic - Universitat de Barcelona, Barcelona, Spain; 2 Universidad Peruana Cayetano Heredia, Instituto de Medicina Tropical Alexander von Humboldt, Lima, Peru; 3 Área de Microbiología Molecular, Centro de Investigación Biomédica de La Rioja (CIBIR), Logroño, Spain

## Abstract

MexAB-OprM and MexEF-OprN are *Pseudomonas aeruginosa* efflux pumps involved in the development of antibiotic resistance. Several studies developed with laboratory strains or using a few clinical isolates have reported that the regulation system of MexEF-OprN is involved in the final levels of MexAB-OprM expression. Therefore, this study was aimed to determine the interplay between MexAB-OprM and MexEF-OprN in 90 out of 190 *P. aeruginosa* clinical isolates with an efflux pump overexpression phenotype. Regarding *oprD*, 33% (30/90) of isolates displayed relevant modifications (RM) defined as frameshift or premature stop, both related to carbapenem resistance. On the other hand, 33% of the isolates displayed RM in *nalC*, *nalD* or *mexR*, which were significantly associated with multidrug resistance (MDR), non-susceptibility to carbapenems, OprD alterations and strong biofilm production. Meanwhile, the RM in MexS were associated with presence of pigment (*p* = 0.004). Otherwise, when all the regulators were analysed together, the association between RM in MexAB-OprM regulators and MDR was only significant (*p* = 0.039) when *mexS* was the wild type. These data show the modulatory effect of MexEF-OprN on MexAB-OprM in a clinical population of *P. aeruginosa*. Further studies may contribute to design of novel molecules acting on this interplay to fight against antimicrobial resistance.

## Introduction

*Pseudomonas aeruginosa* is an opportunistic human pathogen characterised by intrinsic resistance to a variety of antimicrobial agents. This property results from the interplay between drug efflux systems and the low outer membrane permeability of this microorganism^1–4^. *P. aeruginosa* possesses at least 12 structural genes for multidrug efflux pumps belonging to the resistance – nodulation – cell division (RND) family of transporters^2^. Of these, MexAB-OprM, MexCD-OprJ, MexEF-OprN and MexXY-OprM efflux pumps have shown to be of clinical relevance^3,5^. The MexAB-OprM efflux system contributes to the intrinsic resistance of this organism to quinolones, tetracycline, chloramphenicol, novobiocin, macrolides and β-lactams, and its overexpression confers cross-resistance or reduced susceptibility to several antibiotics^2,6^. In addition, it has been reported that MexAB-OprM exports quorum-sensing mediators such as acylhomoserine lactones including *N*-butyryl-L-homoserine lactone (C4-HSL), which induce the production of virulence factors, including proteases, rhamnolipids, exotoxin A, exoenzyme S, and pyocyanin^7^. On the other hand, the MexEF-OprN system is not expressed during growth, and under laboratory conditions it is expressed in *nfxC* multidrug-resistant mutants^2^.

The presence of mutations in MexR, NalC and NalD repressors of MexAB-OprM up-regulate its expression^8,9^, whereas MexEF-OprN expression is enhanced by a positive regulator, MexT, and impaired by MexS expression^7^. In addition, MexT down-regulates *oprD*, the gene encoding the porin OprD which is used by imipenem for cell entry^10,11^.

Although, the concomitant overexpression of both efflux systems have previously been described in several *P. aeruginosa* clinical isolates, suggesting alternative regulation pathways^12,13^, the regulation system of MexEF-OprN is also involved in the final levels of MexAB-OprM expression^1^. Thus, it has been proposed that MexT down-regulates MexAB-OprM^7,14^. Nonetheless, further studies by Richardot *et al*.^15^ have shown that equivalent levels of *mexT* expression may or not drive to down-regulation of the *mexB* gene. This *mexB* down-regulation was associated with overexpression of *mexE* related to the lack of functionality of *mexS*, suggesting a more complex interrelation between the two efflux pumps^15^. In this sense, the NfxC type mutants (overexpressing MexEF-OprN) exhibit low-level production of MexAB-OprM and virulence factors including a lower ability of biofilm formation^15,16^.

This inverse relationship between the expression of MexAB-OprM and the expression of MexEF-OprN could reflect an overlapping of antimicrobial substrates or similar cell-associated extruded products by any of these systems^17^. Nonetheless, this finding may also cause differences in virulence, antimicrobial resistance or specific properties^1^.

Overall, several studies have proposed the interaction between the two efflux systems^2,7,16^, however most of them have mainly been developed in laboratory strains or a few clinical isolates. The purpose of this study was to determine the interplay between MexAB-OprM and MexEF-OprN in antimicrobial resistance, the *oprD* gene, biofilm formation, swarming motility and pigment in a wide variety of clinical isolates of *Pseudomonas aeruginosa* from two Peruvian hospitals.

## Results and Discussion

### Susceptibility to levofloxacin and efflux pump overexpression (EPO) phenotype

Overall, 58% (110/190) of the isolates were non-susceptible to levofloxacin (LVX), with no differences between the two hospitals [Hospital Arzobispo Loayza, (HAL) and Hospital Nacional Cayetano Heredia (HNCH)]; 55% (62/112) in HNCH and 62% (48/78) in HAL. All isolates grew in the presence of phenylalanine-arginyl ß-naphthylamide (PAβN). The EPO phenotype was observed in 47% (90/190) of the isolates, with similar values in HNCH and HAL [45% (50/112), 51% (40/78) respectively].

**Figure 1:**
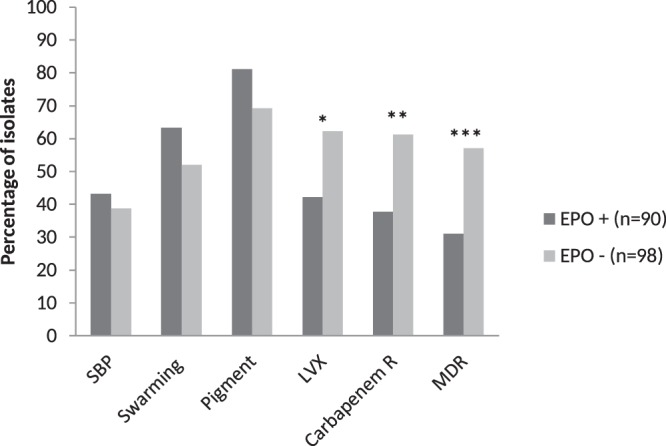
Association of EPO phenotype with biofilm formation, swarming, pigment and antibiotic resistance. SBP: Strong biofilm producer; Swarming: Microorganisms showing swarming motility; Pigment: Presence of pigment; LVX: Resistance to levofloxacin; Carbapenem: resistance to carbapenems; MDR: Multidrug resistance**;** EPO: Efflux Pump Overexpression. In 2 isolates the MIC_I_ was >256 mg/L while the MIC_PABN_ was 256 mg/L, therefore the EPO phenotype was not determined. *p = 0.0093; **p = 0.0013; ***p = 0.006.

The present data demonstrate an inverse relationship between multidrug resistance (MDR) and EPO (*p* = 0.0006). In addition, the EPO phenotype was associated with LVX and carbapenem susceptible isolates (*p* = 0.0093 and 0.0013, respectively) (Fig. 1). Most of the *P. aeruginosa* RND-efflux pumps may extrude fluoroquinolones. Therefore, increases in their activity (either that of an efflux pump alone or of two or more concomitantly) may be easily detected using a fluoroquinolone such as LVX together with an efflux pump inhibitor (EPI)^1^. Nonetheless, the present results agree with a limited effect of efflux pumps, by itself and in the absence of other mechanisms, on the change of the clinical strain classification from Susceptible to Intermediate / Resistant^1^. Additionally, these results may reflect the different substrate affinities presented by different efflux pumps^1^ and the different final effect on minimum inhibitory concentration (MIC) levels related to each *P. aeruginosa* efflux pump.

### Mutations in *oprD* gene

The *oprD* gene was sequenced in all the isolates showing the EPO phenotype. Overall, 67% (60/90) of isolates displayed amino acid changes or deletions which did not affect the OprD frameshift. Thus, 7 isolates showed sequences identical to the *P. aeruginosa* PAO1 strain, 35% (21/60) isolates showed punctual mutations, and 53% (32/60) of the isolates displayed the amino acid deletions S_373_/G_383_. These amino acid deletions were presented in addition to several punctual mutations including V_127_L, E_185_Q, P_186_G, V_189_T, E_202_Q, I_210_A, E_230_K, S_240_T, N_262_T, T_276_A, A_281_G, K_296_Q, Q_301_E, R_310_E/G, G_312_R, A_315_G, K_347_M, V_359_L, S_403_A, Q_424_E as well as a series of changes between amino acid 372 and 383 (_372_V-DSSSSYAGL-_383_). Eighteen additional isolates presented these mutations and also possessed relevant modifications. Overall, punctual mutations were presented conforming sets (Table 1). Of these sets, those involving E_202_Q, I_210_A, E_230_K, S_240_T, N_262_T, A_267_S, A_281_G, K_296_Q, Q_301_E, R_310_G, (±V_352_I), V_359_L, (±Q_424_R) + _372_V-DSSSSYAGL-_383_ (32 isolates, pattern A), T_103_S, K_115_T, F_170_L, E_185_Q, P_186_G, V_189_T, R_310_E, A_315_G, (±G_425_A) (19 isolates, pattern B) and V_127_L, E_185_Q, P_186_G, V_189_T, E_202_Q, I_210_A, E_230_K, S_240_T, N_262_T, T_276_A, A_281_G, K_296_Q, Q_301_E, R_310_E, (±G_312_R), A_315_G, L_347_M, S_403_A, Q_424_E + _372_V-DSSSSYAGL-_383_ (18 isolates, pattern C) were the most frequently detected. It has been described that these types of mutations have no effect on the development of resistance to carbapenems, including several of the most frequently found in present isolates such as T_103_S, K_115_T, F_170_L, E_185_Q, P_186_G, V_189_T, R_310_E, A_315_G, G_425_A (15 isolates) or T_103_S, K_115_T, F_170_L (4 isolates) in OprD^18^. Furthermore, the presence of the alteration _372_V-DSSSSYAGL-_383_, shortening loop L7, has been related to increased susceptibility to meropenem^19,20^; therefore, these types of alterations were classified as “irrelevant modifications” when presented alone. The presence of a potential association has been suggested between specific amino acid substitutions in OprD and MLST profiles, observing a series of amino acid deletions plus a set of punctual mutations on analysing 12 isolates belonging to the ST111^21^. This pattern of OprD amino acid substitutions was almost concordant with the present pattern C, having also strong similarities with pattern A. Therefore, despite the absence of specific MLST determinations, the present results suggest the relevant presence of this high risk *P. aeruginosa* clone in the area. In a previous study^22^, the clonal relationships among these isolates was established, observing a high diversity (72 different clonal patterns). Nonetheless, 72.2% (13/18) of the isolates classified within pattern C, suggestive of belonging to ST111 were from HCNH while the remaining 27.7% (5/18) were from HAL, accounting for 26% and 12.5% of the isolates analysed from each hospital. This finding is in accordance with Kim *et al*.^21^ who described differences in the prevalence of ST111 between different hospitals from the same area. In addition, another common set of amino acid substitutions (pattern B) very similar to those reported by Kim *et al*. ^21^ for *P. aeruginosa* ST298 and ST308 was also detected in 19 isolates.

**Table 1:** Modifications in the *oprD* gene of isolates with the EPO phenotype.

Gene sequence	Type of Modification^a^	Total(n = 90)	HNCH(n = 50)	HAL(n = 40)	Modifications (N)	Carbapenem		
						R (n = 28)	S (n = 56)	*p*
*oprD*	No mutation	7	5	2	—	0	7	
	Amino acid substitution	21	9	12	**T**_**103**_**S, K**_**115**_**T, F**_**170**_**L, E**_**185**_**Q, P**_**186**_**G, V**_**189**_**T, R**_**310**_**E, A**_**315**_**G, G**_**425**_**A (15)**^b^/T_103_S, K_115_T, F_170_L, E_185_Q, P_186_G, V_189_T, G_307_D (1)/T_103_S, K_115_T, F_170_L (4)/V_127_L (1).	3	17	
	Amino acid deletion and substitution	32	17	15	E_202_Q, I_210_A, E_230_K, S_240_T, N_262_T, A_267_S, A_281_G, K_296_Q, Q_301_E, R_310_G, V_359_L, Q_424_R **+**_372_V-DSSSSYAGL-_383_ (21)/**V**_**127**_**L, E**_**185**_**Q, P**_**186**_**G, V**_**189**_**T, E**_**202**_**Q, I**_**210**_**A, E**_**230**_**K, S**_**240**_**T, N**_**262**_**T, T**_**276**_**A, A**_**281**_**G, K**_**296**_**Q, Q**_**301**_**E, R**_**310**_**E, G**_**312**_**R, A**_**315**_**G, L**_**347**_**M, S**_**403**_**A, Q**_**424**_**E +**_**372**_**V-DSSSSYAGL-**_**383**_ (**11**)^c^.	2	30	
	Amino acid insertion	0	0	0	—	0	0	
								<0.0001^d^
	**Frameshift**	12	3	9	ins_nt1087_ (A) + T_103_S, K_115_T, F_170_L (2)/ins_nt1201–1205_ (GTCCA) + **T**_**103**_**S, K**_**115**_**T, F**_**170**_**L, E**_**185**_**Q, P**_**186**_**G, V**_**189**_**T, R**_**310**_**E, A**_**315**_**G (4)**^b^/ins_nt941–942_(GC) (2)/ins_nt678_ (G) + **V**_**127**_**L, E**_**185**_**Q, P**_**186**_**G, V**_**189**_**T, E**_**202**_**Q, I**_**210**_**A, E**_**230**_**K, S**_**240**_**T, N**_**262**_**T, T**_**276**_**A, A**_**281**_**G, K**_**296**_**Q, Q**_**301**_**E, R**_**310**_**E, A**_**315**_**G, L**_**347**_**M, S**_**403**_**A, Q**_**424**_**E +**_**372**_**V-DSSSSYAG L-**_**383**_ **(1)**^c^/ins_nt605–609_ (CAACA) + E_202_Q, I_210_A, E_230_K, S_240_T, N_262_T, A_267_S, A_281_G, K_296_Q, Q_301_E, R_310_G, V_352_I, V_359_L + _372_V-DSSSSYAGL-_383_ (3).	7	0	
	**Stop**	14	12	2	W_65_* + **V**_**127**_**L, E**_**185**_**Q, P**_**186**_**G, V**_**189**_**T, E**_**202**_**Q, I**_**210**_**A, E**_**230**_**K, S**_**240**_**T, N**_**262**_**T, T**_**276**_**A, A**_**281**_**G, K**_**296**_**Q, Q**_**301**_**E, R**_**310**_**E, A**_**315**_**G, L**_**347**_**M, S**_**403**_**A, Q**_**424**_**E + **_**372**_**V-DSSSSYAGL-**_**383**_ **(6)**^c^/Y_49_* + E_202_Q, I_210_A, E_230_K, S_240_T, N_262_T, A_267_S, A_281_G, K_296_Q, Q_301_E, R_310_G, V_359_L + _372_V-DSSSSYAGL_383_- (8).	14	0	
	**No amplification**	4	4	0	—	2	2	

HNCH: Hospital Nacional Cayetano Heredia, HAL: Hospital Arzobispo Loayza; N: Number; ins_nt_: nucleotide insertion; *codon STOP. The slanted line (/) separates different patterns of modifications.

Carbapenems ^R^ are isolates showing resistance or intermediate susceptibility to both imipenem and meropenem, while carbapenem S, are those isolates exhibiting susceptibility to both carbapenems. Six isolates with discordant resistance/susceptibility patterns among imipenem and meropenem were not included in either of the 2 columns.

The amino acid changes located after a stop or frameshift are numbered following the sequence of the wild type strain without considering the presence of this stop or frameshift, and therefore do not represent the protein produced and are only reported for facilitating epidemiological interpretations.

^a^In bold are marked relevant modifications.

^b^Pattern B, very similar to the amino acid changes observed in ST298/ST308^21^.

^c^Pattern C, very similar to the amino acid changes observed in ST111^21^.

^d^Significant differences in carbapenem resistance levels between isolates possessing relevant and irrelevant modifications.

On the other hand, alterations affecting porin functionality (lack of gene, premature STOPs or frameshifts) were classified as “relevant modifications”. Overall, 33% (30/90) of the isolates showed relevant modifications; 40% (12/30) presenting frameshifts by base pair insertions and 47% (14/30) possessing premature stops in amino acid codons 65 and 49, and in four isolates no PCR amplification was obtained. Overall, the relevant modifications were strongly associated with carbapenem non-susceptible isolates (*p* < 0.0001) (Table 1). This finding correlates with other studies showing that carbapenem resistance is mainly associated with inactivation of the *oprD* gene^21^. In the present study, 33% of our isolates showed functional alterations containing mainly frameshifts and premature stops in the gene leading to truncated proteins, similar to the results reported by Kim *et al*.^21^. In addition, although PCR impairment due to DNA polymorphisms cannot be ruled out, the lack of amplification of *oprD* in four isolates could be explained by the presence of an insertion sequence (IS). In this sense the presence of disruption of the *oprD* gene by different ISs including the IS*Pa27*, IS*Pa45*, IS*Pa46*, IS*Pa47*, IS*Pa133*, IS*Pa1328*, IS*Pa1635*, IS*Pst12* or IS*Ppu*21 has previously been shown^23,24^.

### Regulatory genes studies in MexAB-OprM

Three MexAB-OprM regulators were analysed. Regarding the *mexR* gene, 47% (42/90) of the isolates showed punctual mutations leading to amino acid changes, being V_126_E [98%(41/42)] the most frequent; 43% (39/90) of the isolates did not have any modification, and the remaining 10% (9/90) of isolates repeatedly did not amplify by PCR assay. Difficulties in PCR amplification may have been due to the presence of polymorphisms in the primers annealing regions, or the presence of internal DNA sequence modifications resulting in specific DNA conformation which impaired PCR amplification. However, the non amplification of the *mexR* gene was probably associated with the presence of a disrupting IS, such as IS*21*, which has previously been described as breaking *mexR* and leading to an increased transcription of the *mexAB-oprM* operon^25^.

In relation to the *nalC* gene, 87% (77/90) of the isolates showed punctual mutations, being G_71_E (76/77) and S_209_R (67/77) the most frequent. In addition, one isolate showed a 10 base pair deletion from C_234_ to G_243_. Regarding *nalD*, 71% (64/90) of the isolates did not show modifications, and 20% (18/90) showed relevant modifications, being two base pair deletions (Δ_nt397–398_) the most frequent in 39% (7/18) of the isolates (Table 2). Similar to our results, the presence of deletions in *nalC* has been previously shown in *P. aeruginosa* isolates^26^. In addition, Haenni *et al*.^27^ described different alterations in the *nalD* gene, including a gene disruption mediated by IS*As2* in isolates of *P. aeruginosa*. This finding may have occurred in two of our isolates that did not amplify this gene.

**Table 2:** Modifications in efflux pump regulators in isolates with the EPO phenotype.

Gene sequence	Type of Modification	Total (n=90)	HNCH (n=50)	HAL (n=40)	Modifications
**MexAB-OprM regulators**					
*mexR*	No mutation^a^	39	21	18	—
	Amino acid substitution	42	23	19	**V**_**126**_**E (39)**/L_131_P (1)/V_126_E, L_131_P (1)/V_126_E, P_143_L (1)
	Amino acid deletion	0	0	0	
	Amino acid insertion	0	0	0	—
	**Frameshift**	**0**	**0**	**0**	—
	**No amplification**	**9**	**6**	**3**	—
	**Stop**	**0**	**0**	**0**	—
*nalC*	No mutation	10	4	6	—
	Amino acid substitution	77	43	34	**G**_**71**_**E, S**_**209**_**R (54)**/**G**_**71**_**E, A**_**145**_**V, S**_**209**_**R (5)**/**G**_**71**_**E, S**_**209**_**R, P**_**210**_**L (5)**/**G**_**71**_**E (4)**/ G_71_E, A_186_T (4)/G_71_E, D_79_R, S_209_R (2)/G_71_E, H_150_Q, M_151_P, D_152_E, E_153_R, (1)/G_71_E, E_153_Q (1)/S_209_R (1)
	Amino acid deletion	0	0	0	—
	Amino acid insertion	0	0	0	—
	**Frameshift**	**1**	**1**	**0**	Δ_nt234–243_ + **G**_**71**_**E**
	**No amplification**	**2**	**2**	**0**	—
	**Stop**	**0**	**0**	**0**	—
*nalD*	No mutation	64	35	29	—
	Amino acid substitution	8	6	2	**T**_**188**_**A (4)**/L_44_P (1)/D_187_S (1)/L_194_R (1)/Q_134_H, Q_142_H, A_145_P, D_147_H, E_148_K, C_149_R, H_154_P, R_160_K, D_176_E, D_185_Y, G_206_S, S_209_I (1)
	Amino acid deletion	0	0	0	—
	Amino acid insertion	0	0	0	—
	**Frameshift**	**16**	**8**	**8**	Δ_nt397–398_ (7)/Δ_nt263–279_ (6)/Δ_nt391_ (2)/Δ_nt451–461_ (1)
	**No amplification**	**2**	**1**	**1**	
	**Stop**	**0**	**0**	**0**	—
**MexEF-OprN regulators**					
*mexT*	No mutation^b^	82	47	35	—
	Amino acid substitution	3	1	2	D_290_E (1)/V_269_E (1)/G_148_A, G_238_R, A_249_P (1)
	Amino acid deletion	0	0	0	—
	Amino acid insertion	0	0	0	—
	**Frameshift**	**0**	**0**	**0**	—
	**No amplification**	**5**	**2**	**3**	—
	**Stop**	**0**	**0**	**0**	—
*mexS*	No mutation^b^	50	31	19	—
	Amino acid substitution	3	1	2	V_73_A (2)/ G_224_S (1)
	Amino acid deletion	0	0	0	—
	Amino acid insertion	0	0	0	—
	**Frameshift**	**0**	**0**	**0**	—
	**No amplification**	**37**	**18**	**19**	—
	**Stop**	**0**	**0**	**0**	—

HNCH: Hospital Nacional Cayetano Heredia, HAL: Hospital Arzobispo Loayza,

In bold are both marked relevant modifications as well as amino acid change patterns previously described by Quale *et al*.^26^. The slanted line (/) separates different patterns of modifications.

The symbol Δ_nt_ means nucleotide deletion being noted the first and last nucleotides deleted.

The amino acid changes located after a frameshift are numbered following the sequence of the wild type strain without considering the presence of this frameshift, and therefore do not represent the protein produced and are only reported for facilitating epidemiological interpretations.

In parenthesis, the number of each specific alteration or combined alterations described. In all cases if a relevant modification was found the sequences are listed in this section, irrespectively of the remaining modifications detected.

^a^In isolates in which no mutation was observed, the MexAB-OprM regulator sequences were identical to those of *P. aeruginosa* PAO1 (GenBank: AE004091.2).

^b^In all isolates in which PCR amplification was obtained, the *mexS* and *mexT* genes were identical to those of *P. aeruginosa* PA14 (GenBank: CP000438).

**Figure 2:**
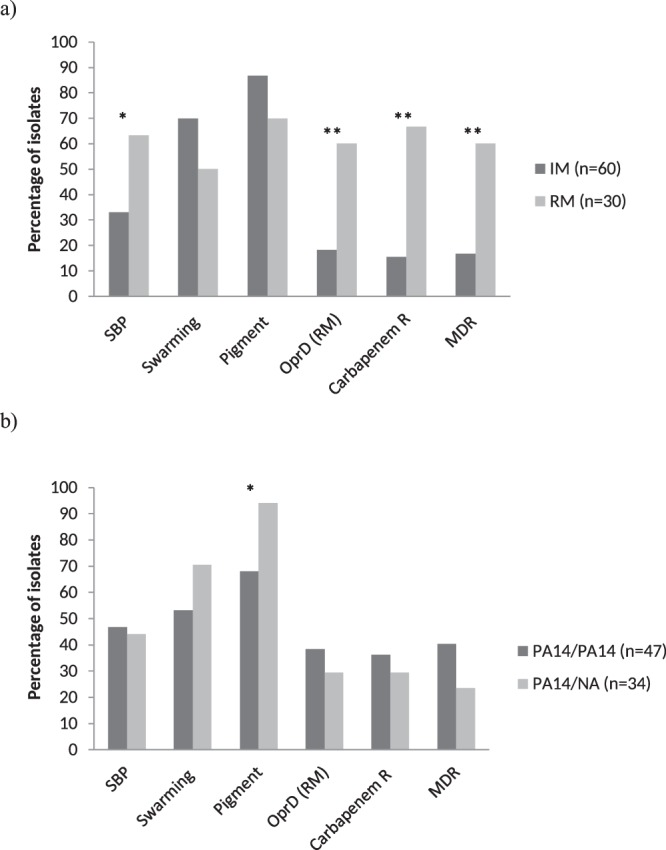
Association of MexAB-OprM regulators and MexEF-OprN regulators with biofilm formation, swarming, pigment, *oprD* gene and antibiotic resistance. SBP: Strong biofilm producer; Swarming: Microorganisms showing swarming motility; Pigment: Presence of pigment; OprD (RM): Presence of relevant modifications in OprD (frameshifts + premature STOPs + no amplification); Carbapenem: resistance to carbapenems; MDR: Multidrug resistance. (**a**) MexAB-OprM regulators. IM: Irrelevant modifications (amino acid substitution + amino acid insertions + amino acid deletions); RM. Relevant Modifications (frameshifts + premature STOPs + no amplification); *p: 0.006; **p < 0.0001. (**b**) MexEF-OprN regulators. PA14: Sequence identical to PA14; NA: no amplification. *p: 0.004. Only the isolates presenting the genotypes PA14/PA14 (47 isolates) and NA/PA14 (34 isolates) were analysed.

It has been described that genetic events such as frameshifts, disruptions or premature stops, which lead to loss of functionality of *nalC*, *nalD* or *mexR* are expected to up-regulate the *mexAB-oprM* operon^25–28^, and therefore were considered as relevant modifications. In the present study, 33% (30/90) of the isolates displayed relevant modifications in the *mexR*, *nalC* or *nalD* genes which were significantly associated with MDR (*p* < 0.0001), carbapenem non-susceptible isolates (*p* < 0.0001) and relevant modifications of the *oprD* gene (*p* < 0.0001). In addition, these relevant modifications were significantly associated with strong biofilm producer (SBP) isolates (*p* = 0.006) [Fig. 2a].

Meanwhile, several of the amino acid changes detected, including some of the most frequently found, such as V_126_E detected in *mexR* in 39 isolates or G_71_E, S_209_R or G_71_E, A_145_V, S_209_R detected in *nalC* in 54 isolates and 5 isolates, respectively, have previously been described in isolates not displaying MexAB-OprM overexpression^26,29^. Thus, *mexR*, *nalC* and *nalD* amino acid changes were classified as “irrelevant modifications”.

To fully determine the role of the modifications detected in the final expression levels of the *mexA* gene, 20 isolates carrying different modifications in efflux-pump regulator genes were selected. The results showed that 8 out of 11 isolates (1084, 1085, 1086, 1089, 1090, 1093, 1094, 1096) carrying relevant modifications in *mexR*, *nalC* or *nalD* presented relative *mexA* expression levels of 1.61 to 5.10 compared to PAO1. Meanwhile, only 2 out of 9 isolates (1082 and 1092) carrying irrelevant modifications presented expression levels higher than PAO1 (1.51 to 3.58) (Table 3). Therefore, on analysing the selected isolates together it was observed that relevant modifications were associated with higher *mexA* expression levels (*p* = 0.02).

**Table 3:** Expression levels of *mexA* and *mexE* in *Pseudomonas aeruginosa* carrying specific alterations at MexAB-OprM/MexEF-OprN regulators.

Isolates	MexAB-OprM regulators			MexEF-OprN regulators		Transcript Level	
	*nalC*	*nalD*	*mexR*	*mexS*	*mexT*	*mexA* ^a^	*mexE* ^a^
1082	G_71_E, S_209_R	WT	WT	WT	**NA**	3.58	1.41
1083	WT	WT	WT	WT	WT	0.13	0.71
1084	G_71_E, A_145_V, S_209_R	**NA**	WT	**NA-c**	WT	4.36	0.53
1085	G_71_E, S_209_R	**Δ**_**nt451–461**_	WT	**NA-c**	WT	1.84	0.45
1086	G_71_E, S_209_R	**Δ**_**nt397–398**_	V_126_E	WT	WT	5.10	7.92
1087	G_71_E, A_145_V, S_209_R	WT	V_126_E	**NA-c**	WT	0.97	1.98
1088	G_71_E, A_145_V, S_209_R	WT	V_126_E	**NA-c**	G_148_A, G_238_R, A_249_P	0.80	0.64
1089	G_71_E, S_209_R	**Δ**_**nt397–398**_	V_126_E	WT	WT	1.80	1.41
1090	G_71_E, S_209_R	**Δ**_**nt397–398**_	V_126_E	WT	WT	1.90	0.83
1091	G_71_E, S_209_R	WT	WT	**NA-c**	**NA**	0.34	0.37
1092	G_71_E, S_209_R	Q_134_H, Q_142_H, A_145_P, D_147_H, E_148_K, C_149_R, H_154_P, R_160_K, D_176_E, D_185_Y, G_206_S, S_209_I	V_126_E	WT	WT	1.51	0.53
1093	G_71_E, S_209_R	**Δ**_**nt397–398**_	V_126_E	WT	WT	3.74	0.83
1094	**Δ**_**nt 234–243**_	WT	WT	**NA-b**	WT	1.61	1.11
1095	G_71_E, S_209_R	WT	NA	**NA-b**	WT	1.04	8.92
1096	G_71_E, S_209_R	**Δ**_**nt391**_	V_126_E	G_224_S	WT	1.84	0.13
1097	G_71_E, S_209_R	**Δ**_**nt391**_	WT	**NA-b**	WT	0.38	0.91
1098	G_71_E, S_209_R, P_210_L	T_188_A	WT	WT	WT	0.85	0.96
1099	G_71_E, A_145_V, S_209_R	WT	V_126_E	WT	WT	1.26	0.76
1100	G_71_E, S_209_R	WT	NA	WT	WT	0.80	4.81
1101	WT	WT	WT	**NA-c**	WT	0.09	1.16

**Figure 3:**
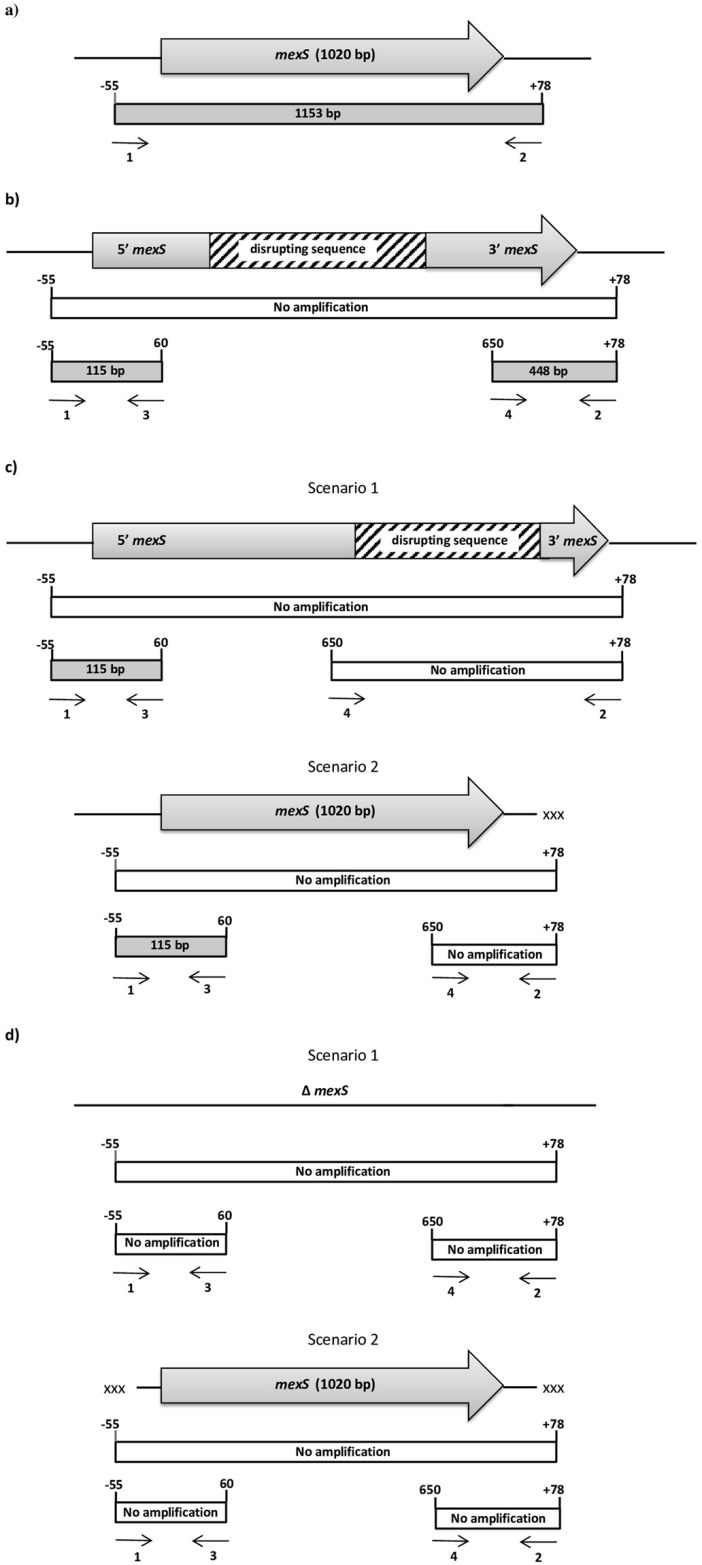
PCR strategy used in the analysis of *mexS* gene. In all figure sections is first presented the scenario which explain the obtained results, and just below the scheme of the PCR reactions. When in grey a positive amplification was obtained, and the amplicon size is within the rectangle, when in blank, no amplification was obtained. In all cases the primers used are represented by thin arrows and numbered following the same numeration presented in Table 4. All positions arbitrarily refer to the first base of the *mexS* gene. The figure is not made to scale. Furthermore, in scenarios b and c, the presence of internal modifications in DNA sequence (affecting or not *mexS* functionality) may lead to DNA secondary structures which obstacle PCR amplification. (**a**) PCR amplification of *mexS* gene. (**b**) No amplification of *mexS* gene and amplification of N- and C-terminal regions. (**c**) No amplification of *mexS* gene and amplification of N-terminal regions. Two scenarios are considered. Scenario 1: a DNA sequence (represented with a weft filling rectangle) disrupt *mexS* after base position 650, allowing the amplification on the N-terminal region but avoiding that of full *mexS* gene as well as that of the C-terminal region. Scenario 2: a polymorphism (represented with “xxx”) avoid the annealing of primer 2. (**d**) No amplification of Mex S gene and no amplification of N- and C- terminal regions. Two scenarios are considered. Scenario 1: the *mexS* gene has been deleted or is absent. Scenario 2: polymorphisms are present in both annealing position of primers 1 and 2. If this late option was right, the most probable is the presence of additional differences in the sequence.

WT: wild type (*nalC*, *nalD*, *mexR* identical to that of PAO1; *mexS* and *mexT* identical to those of PA14); NA: No amplification; NA-b: In addition to no amplification of *mexS*, no amplification of either *mexS* N- and C-terminal regions (see panel b of Fig. 3); NA-c: In addition to no amplification of *mexS*, no amplification of the *mexS* C-terminal region (see panel c of Fig. 3).

Relevant modifications are marked in bold.

^a^Relative gene expression was calculated by 2^−ΔΔCT^ method. The *rpsL* gene was used as reference*, P. aeruginosa* PAO1 strain as calibrator in *mexA* gene (value = 1) and *P. aeruginosa* PA14 strain was used as calibrator in *mexE* gene (value = 1). Expression levels increases ranging between 1.5 and 2-fold were considered as borderline, and those increases in the expression levels >2 were classified as overexpression^3,13^.

Previous studies have reported that the MexAB-OprM efflux system contributes to the intrinsic resistance of *P. aeruginosa* to several antimicrobials such as quinolones, chloramphenicol and most β-lactams and its overexpression contributes to MDR phenotypes^1,2,30^. Similarly, in the present study, isolates with relevant modifications in any of the MexAB-OprM regulators analysed were significantly associated with MDR. This finding is in accordance with the above mentioned different effect on final resistance levels of the overexpression of different efflux pumps, contributing to explain the observed lack of association between MDR (or LVX) and overall EPO, highlighting the role of MexAB-OprM in the development of antibiotic resistance.

In addition, we observed that the presence of relevant modifications in the MexAB-OprM regulators efflux system was significantly associated with reduced susceptibility to carbapenems (*p* < 0.0001) and relevant modifications in the *oprD* gene (p < 0.0001) (Table 4). This is in accordance with the role of the loss or low expression of OprD combined with the overexpression of this efflux system in carbapenem resistance mechanisms in *P. aeruginosa* isolates^18,30–34^. Interestingly, the presence of relevant modifications in MexAB-OprM regulators and OprD lack of functionality would seem to be independent phenomena. Nonetheless, the association observed may reflect an external pressure (e.g.: antibiotic consumption) affecting both systems. Furthermore, in agreement with previous studies in which overproduction of MexAB-OprM was correlated with biofilm production^35^, our results showed that the presence of relevant modifications in MexAB-OprM regulators was significantly associated with strong biofilm producer isolates.

**Table 4:** Primers used for PCR amplification.

Amplified product	Primers	F1	Sequence (5′ → 3′)	Amplicon size (bp)	Annealing Temperature	Reference
**Efflux pump regulators and** ***oprD*** **gene**						
*mexR*	mexR - F		ATT CGC CAG TAA GCG GAT AC	1020	60 °C	^9^
	mexR - R		GGA TGA TGC CGT TCA CCT G			
*nalC*	nalC - F		TCA ACC CTA ACG AGA AAC GCT	814	69 °C	^9^
	nalC - R		TCC ACC TCA CCG AAC TGC			
*nalD*	nalD - F		GCG GCT AAA ATC GGT ACA CT	789	54 °C	^9^
	nalD - R		ACG TCC AGG TGG ATC TTG G			
*mexT*	mexT - F		TGC ATC ACG GGG TGA ATA AC	1398	60 °C	^9^
	mexT - R		GGT AGC GCC AGG AGA AGT G			
*mexS*	mexS - F		ATA CAG TCA CAA CCC ATG A	1153	60 °C	^9^
	mexS - R		TCA ACG ATC TGT GGA TCT			
*oprD*	oprD - F		GGC AGA GAT AAT TTC AAA ACC AA	1384	60 °C	^41^
	oprD - R		GTT GCC TGT CGG TCG ATT AC			
**N- and C- terminal regions of** ***mexS*** ^**a**^						
*N-terminal*	mexS – F	1	ATA CAG TCA CAA CCC ATG A	115	60 °C	^9^
	mexS – N	3	CTC TTC GCA TTT GAG GAC C			This study
*C-terminal*	mexS – C	4	CAT CCT CGA CGA ATT GGG	448	60 °C	This study
	mexS – R	2	TCA ACG ATC TGT GGA TCT			^9^
**qRT-PCR analysis of efflux pumps**						
*mexA*	mexA - F		GGC GAC AAC GCG GCG AAG G	203	60 °C	^13^
	mexA - R		CCT TCT GCT TGA CGC CTT CCT GC			
*mexE*	mexE - F		TCA TCC CAC TTC TCC TGG CGC TAC C	150	60 °C	^13^
	mexE - R		CGT CCC ACT CGT TCA GCG GTT GTT CGA TG			
*rpsL*	rpsL - F		CGG CAC TGC GTA AGG TAT GC	212	60 °C	^42^
	rpsL - R		CGT ACT TCG AAC GAC CCT GCT			

F1: Correspondence with Fig. 1; bp: base pair; F: Forward; R: reverse.

^a^Primers used to amplify the N- and C-terminal regions, respectively.

### Regulatory gene studies in MexEF-oprN

No modification in the *mexT* gene was observed in 90% (81/90) of the isolates (being identical to the sequence of the PA14 reference strain) and 3.3% (3/90) of the isolates presenting amino acid changes [D_290_E (1)/V_269_E (1)/G_148_A, G_238_R, A_249_P (1)]. The remaining 5.5% (5/90) of the isolates did not amplify the *mexT* gene. Meanwhile, 59% (53/90) of the isolates showed N_249_ in the *mexS* gene, being identical to the sequence of PA14. Only 3 of these isolates carried a single amino acid change in positions V_73_A (2) and G_224_S (1). All were considered as fully functional and able to inhibit the expression of MexEF-OprN (Table 2)^15^. The remaining 41% (37/90) of the isolates did not amplify the *mexS* gene (Table 2). Of these 37 isolates, it was possible to amplify the *mexS* gene N- and C-terminal regions in 11 isolates, while in 23 isolates only the N-terminal region was amplified. In 3 isolates neither the N-nor C-terminal regions was amplified (Fig. 3).

Only two combinations of the *mexS* and *mexT* genes were further analysed: i) the *mexS* and *mexT* genes identical to those of PA14 (47 isolates), and ii) the non amplified *mexS* gene and *mexT* gene identical to that of PA14 (34 isolates). The remaining scenarios were not further analysed due to the small numbers of isolates presenting the required characteristics (9 isolates). The second combination was significantly associated with the presence of pigment (*p* = 0.004) (Fig. 2b).

As mentioned above, amino acid changes both in *mexS* and *mexT* were classified as irrelevant modifications. Nonetheless, a direct effect of the amino acid substitutions detected on the functionality of these regulators cannot be ruled out^15^. Thus, the analysis of isolate 1096 presenting the amino acid change G_224_S showed *mexE* expression levels of 0.13, suggesting an enhancement of the inhibitory activity of MexS instead of a loss of MexS function. Meanwhile, in isolate 1088 carrying amino acid changes in MexT (G_148_A, G_238_R, A_249_P), the *mexE* expression values were of 0.64, within the range of isolates 1083, 1090, 1092,1093, 1099 which did not carry alterations in both MexS and MexT.

The *mexE* gene expression analyses showed that no amplification of *mexS* only correlated with *mexE* overexpression in 2 isolates (1087 and 1095). In isolate 1095, in which the expression levels of *mexE* were of 8.92, both N- and C-terminal regions were amplified while in isolate 1087 (*mexE* expression levels of 1.98) a PCR product was only obtained by amplifying the N-terminal region. In both cases, this may have occurred due to the presence of an IS disrupting the *mexS* gene^27^. Meanwhile, in other 6 isolates (1084, 1085, 1088, 1094, 1097, 1101) no deregulation of *mexE* was observed (*mexE* expression levels ranging from 0.53 to 1.16), despite the presence of a fully functional *mexT*, thereby suggesting the presence of polymorphisms in *mexS* primer annealing regions and/or specific internal DNA conformation hampering PCR amplification, although a possible impairment of MexT activity in isolate 1088 related to specific amino acid changes cannot be ruled out. Nonetheless, it should be mentioned that a similar scenario of non *mexE* overexpression in the presence of fully functional MexT and inactive MexS has previously been described^12^. In the remaining isolate analysed (1091) with relevant alterations in *mexS*, the *mexE* expression levels of 0.37 were concordant with the absence of *mexT* amplification, which as mentioned above might be related to the presence of a disrupting internally inserted sequence (Table 3). In this line, Quale *et al*. detected up to 7 isolates showing diminished (arriving to 0) expression of *mexE* in which it was only possible to amplify the initial 462 bp of *mexT*, suggesting major mutations affecting this region^26^. Finally, in isolates 1086 and 1100 a clear overexpression of *mexE* (expression levels of 7.92 and 4.81 respectively) was observed, despite the *mexS* and *mexT* genes being identical to those of PA14 (Table 3). This result shows the role of other regulators in the final expression levels of *mexEF-oprN*. In this line, modifications of the *mvtA* gene have been related to *mexEF-oprN* overexpression^36^.

Different from what was observed on analysing the MexAB-OprM regulators, no association was found between relevant modifications in MexEF-OprN regulators and MDR or the presence of an *oprD* gene frameshift. The only association observed was present among isolates with MexEF-OprN regulator relevant modifications and the presence of pigment (Fig. 2b). This finding disagrees with the reduced production of virulence factors such as biofilm formation, pyocyanin or rhamnolipids among others, in isolates overexpressing MexEF-OprN^34,37^. Nonetheless, it should be taken into account that despite the above commented impairment in the production of pyocyanin in isolates overexpressing MexEF-OprN, a role of MexEF-OprN in the excretion of intermediates of pyocyanin biosynthesis has been proposed^38^.

### Interplay of the MexAB-OprM and MexEF-OprN

Previous studies have reported that C4-HSL induces the expression of the *mexAB-oprM* operon directly by binding at the MexR-MexAB-OprM operator-promoter region^7^. It has been reported that the *nfxC* mutant isolates overexpress MexEF-OprN, decreasing the production of C4-HSL^7^, and subsequently those of MexAB-OprM, thereby having a negative effect on MexAB-OprM exported products and homoserine lactone-dependent virulence factors^7^. Likewise, the association between relevant modifications in MexAB-OprM regulators and MDR was only significant (*p* = 0.039) when *mexS* was wild type, and therefore able to exert a negative regulation effect on the expression levels of MexEF-OprN. Furthermore, in 2 out of 3 isolates (isolates 1095 and 1100) in which the presence of relevant modifications in the *mexAB-OprM* regulators did not result in *mexA* overexpression (expression levels of 1.04 and 0.8 respectively), the expression levels of *mexE* were of 4.81 and 8.92 (Table 3).

On the other hand, the final expression levels of MexAB-OprM and MexEF-OprN with isolate 1086 showed increased expression levels of both *mexA* (expression levels of 5.10) and *mexE* (expression levels of 7.92) (Table 3), which agree with the concomitant overexpression of both efflux systems previously described in several *P. aeruginosa* clinical isolates by different authors^12,13^.

Overall, the present data showed a relevant role of modifications leading to the loss of MexR, NalC and NalD functionality in the clinical isolates analysed, which were associated with higher levels of antibiotic resistance and different bacterial virulence including biofilm formation. The effect of these modifications on multidrug resistance levels was significantly higher in the presence of *mexS* amplification, highlighting the modulatory effect of mexEF-OprN overexpression on the final resistance phenotype.

## Methods

### Bacterial strains

We studied a total of 190 isolates of *P. aeruginosa* from clinical samples of patients attended at the HAL (78 isolates) and the HNCH (112 isolates) in Lima (Peru), from December 2012 to June 2013. In all cases only non-duplicated isolates from different patients were included in the study. The isolates were stored at −70 °C in skim milk medium (Oxoid, Hampshire, UK) until use. The clonal relationships, carbapenem susceptibility and MDR levels, biofilm formation, swarming motility and pigment presence were determined in a previous study^22^. In all cases antibiotic susceptibility was classified, according CLSI breakpoints^39^. MDR was defined as resistance to three or more unrelated families of antibiotics (aminoglycosides, β-lactams, fluoroquinolones and polymyxin). The isolates intermediate or resistant to both imipenem and meropenem were classified as “carbapenem resistant”. Throughout the text the term “resistance” englobes resistant and intermediate isolates.

### Efflux pump inhibition test

EPO was established by determining the effect of the EPI PAβN (Sigma Chemical, Co, St. Louis, MO) on the MICs of LVX. Thus, the MIC of LVX was established by the agar dilution method^39^ both with (MIC_PAβN_) 20 µg/ml and without (MIC_I_) of PAβN. An EPO phenotype was defined when MIC_I_/MIC_PABN_ was >2 as previously described^40^. The effect of this concentration of PAβN on the viability of microorganisms was also assessed.

### *oprD* gene amplification

Amplification of the *oprD* gene was performed by PCR (Table 4)^41^. Negative PCRs were performed twice in order to avoid false negative results. In all cases the PCR products were recovered and fully sequenced and compared to the reference strain PAO1 (GenBank: AE004091.2).

### Efflux pumps gene regulators

The primers and PCR amplification conditions of the efflux regulator-encoding genes *mexR*, *nalC*, *nalD*, *mexT* and *mexS* were designed by Solé *et al*.^9^ with slight modifications of the annealing conditions (Table 4). All PCR products were sequenced as above. When the PCR product did not amplify, the assay was performed twice to avoid false negative results. After that, negative PCR were considered to as genes with “relevant modifications”. The *mexR*, *nalC* and *nalD* genes were compared with those of *P. aeruginosa* strain PAO1 (GenBank: AE004091.2). However the *mexT* and *mexS* genes were analysed according to the full functional MexS and MexT of *P. aeruginosa* PA14 (GenBank: CP000438), because PAO1 lacks the functionality of those genes related to the presence of 8 bp insertion resulting in a frameshift in MexT and D_249_ in MexS^15^. To determine the presence of undetected insertions within to the *mexS* gene, a PCR strategy was designed. Briefly, in those isolates in which PCR product was repeatedly not obtained, two new PCR reactions were designed in order to amplify the N- and C-terminal regions, respectively (Table 4).

### Efflux Pump expression

The expression levels of *mexA* and *mexE* were determined in 20 *P. aeruginosa* isolates representative of the different alterations encountered in the regulator genes. mRNA extraction and qRT-PCR were performed following the primers and methodology previously described (Table 4)^13,42^. In all cases, gene expression was normalised *versus rpsL* housekeeping gene and expression levels were indicated as a ratio to the expression level in strain PA01 (*mexA*) or PA14 *(mexE*).

### Statistical analysis

The χ^2^ (Chi square test) was used to determine the presence of significant differences which were considered with a *p* value of ≤0.05. R studio version 3.4.0.was used for all statistical analyses. Resistant and intermediate isolates were classified together as “non-susceptible” for statistical analyses.

### Compliance with ethical standards

The study was approved by the Ethical Committee of the Universidad Peruana Cayetano Heredia (Lima, Peru) and by the Ethical Committee of Hospital Clinic (Barcelona, Spain), and all experiments were performed in accordance with relevant guidelines.

All samples were obtained within routine clinical practice; no personal data was requested or available to researchers.

## References

[1] Li XZ, Plésiat P, Nikaido H (2015). The challenge of efflux-mediated antibiotic resistance in Gram-negative bacteria. Clin.. Microbiol. Rev..

[2] Li XZ, Zhang L, Poole K (2000). Interplay between the MexA-MexB-OprM multidrug efflux system and the outer membrane barrier in the multiple antibiotic resistance of *Pseudomonas aeruginosa*. J. Antimicrob. Chemother..

[3] Poonsuk K, Tribuddharat C, Chuanchuen R (2014). Simultaneous overexpression of multidrug efflux pumps in *Pseudomonas aeruginosa* non-cystic fibrosis clinical isolates. Can. J. Microbiol..

[4] Strateva T, Yordanov D (2009). *Pseudomonas aeruginosa* - a phenomenon of bacterial resistance. J.Med. Microbiol..

[5] Li, X. Z., Elkins, C. A. & Zgurskaya, H. I. Efflux-mediated antimicrobial resistance in bacteria. 651p. (Springer International Publishing, 2016).

[6] Henrichfreise B, Wiegand I, Pfister W, Wiedemann B (2007). Resistance mechanisms of multiresistant *Pseudomonas aeruginosa* strains from Germany and correlation with hypermutation. Antimicrob. Agents. Chemother..

[7] Maseda H (2004). Enhancement of the *mexAB-oprM* efflux pump expression by a quorum-sensing autoinducer and its cancellation by a regulator, MexT, of the *mexEF-oprN* efflux pump operon in *Pseudomonas aeruginosa*. Antimicrob. Agents Chemother..

[8] Poole K (2011). *Pseudomonas aeruginosa*: Resistance to the Max. Front. Microbiol..

[9] Solé M (2015). *In vivo* evolution of resistance of *Pseudomonas aeruginosa* strains isolated from patients admitted to an intensive care unit: mechanisms of resistance and antimicrobial exposure. J. Antimicrob. Chemother..

[10] Köhler T (1997). Characterization of MexE-MexF-OprN, a positively regulated multidrug efflux system of *Pseudomonas aeruginosa*. Mol. Microbiol..

[11] Li H, Luo YF, Williams BJ, Blackwell TS, Xie CM (2012). Structure and function of OprD protein in *Pseudomonas aeruginosa:* from antibiotic resistance to novel therapies. Int. J. Med. Microbiol..

[12] Singh M, Yau YCW, Wang S, Waters V, Kumar A (2017). MexXY efflux pump overexpression and aminoglycoside resistance in cystic fibrosis isolates of *Pseudomonas aeruginosa* from chronic infections. Can J Microbiol..

[13] Tomás M (2010). Efflux pumps, OprD porin, AmpC β-lactamase, and multiresistance in *Pseudomonas aeruginosa* isolates from cystic fibrosis patients. Antimicrob Agents Chemother..

[14] Llanes C (2011). Role of the MexEF-OprN efflux system in low-level resistance of *Pseudomonas aeruginosa* to ciprofloxacin. Antimicrob. Agents Chemother..

[15] Richardot C (2016). Amino acid substitutions account for most MexS alterations in clinical *nfxC* mutants of *Pseudomonas aeruginosa*. Antimicrob. Agents Chemother..

[16] Uwate M (2013). Two routes of MexS-MexT-mediated regulation of MexEF-OprN and MexAB-OprM efflux pump expression in *Pseudomonas aeruginosa*. Microbiol. Immunol..

[17] Poole K (2001). Multidrug efflux pumps and antimicrobial resistance in *Pseudomonas aeruginosa* and related organisms. J. Mol. Microbiol. Biotechnol..

[18] Ocampo-Sosa AA (2012). Alterations of OprD in carbapenem-intermediate and susceptible strains of *Pseudomonas aeruginosa* isolated from patients with bacteremia in a Spanish multicenter study. Antimicrob. Agents. Chemother..

[19] Epp. SF (2001). C-terminal region of *Pseudomonas aeruginosa* outer membrane porin OprD modulates susceptibility to meropenem. Antimicrob Agents Chemother..

[20] Kao C-Y (2016). Overproduction of active efflux pump and variations of OprD dominate in imipenem-resistant *Pseudomonas aeruginosa* isolated from patients with bloodstream infections in Taiwan. BMC Microbiol..

[21] Kim CH (2016). Mutational inactivation of OprD in carbapenem-resistant *Pseudomonas aeruginosa* isolates from Korean hospitals. J. Microbiol..

[22] Horna, G. *et al*. Specific Type IV Pili groups in clinical isolates of *Pseudomonas aeruginosa*. *Int Microbiol*. **In press**10.1007/s10123-018-00035-3 30810940

[23] Estepa V (2017). Characterisation of carbapenem-resistance mechanisms in clinical *Pseudomonas aeruginosa* isolates recovered in a Spanish hospital. Enferm. Infecc. Microbiol. Clin..

[24] Rodríguez-Martínez JM, Poirel L, Nordmann P (2009). Molecular epidemiology and mechanisms of carbapenem resistance in *Pseudomonas aeruginosa*. Antimicrob. Agents Chemother..

[25] Boutoille D (2004). Detection of an IS21 insertion sequence in the *mexR* gene of *Pseudomonas aeruginosa* increasing beta-lactam resistance. FEMS Microbiol. Lett..

[26] Quale J, Bratu S, Gupta J, Landman D (2006). Interplay of efflux system, *ampC*, and *oprD* expression in carbapenem resistance of *Pseudomonas aeruginosa* clinical isolates. Antimicrob. Agents. Chemother..

[27] Haenni M (2017). Resistance of animal strains of *Pseudomonas aeruginosa* to carbapenems. Front. Microbiol..

[28] Cao L, Srikumar R, Poole K (2004). MexAB-OprM hyperexpression in NalC-type multidrug-resistant *Pseudomonas aeruginosa*: identification and characterization of the *nalC* gene encoding a repressor of PA3720-PA3719. Mol. Microbiol..

[29] Ziha-Zarifi I, Llanes C, Köhler T, Pechere JC, Plesiat P (1999). *In vivo* emergence of multidrug-resistant mutants of *Pseudomonas aeruginosa* overexpressing the active efflux system MexA-MexB-OprM. Antimicrob Agents Chemother..

[30] Pan YP, Xu YH, Wang ZX, Fang YP, Shen JL (2016). Overexpression of MexAB-OprM efflux pump in carbapenem-resistant. Pseudomonas aeruginosa. Arch. Microbiol..

[31] Fang ZL (2014). OprD mutations and inactivation in imipenem-resistant *Pseudomonas aeruginosa* isolates from China. Infect. Genet. Evol..

[32] Lee JY, Ko KS (2012). OprD mutations and inactivation, expression of efflux pumps and AmpC, and metallo-β-lactamases in carbapenem-resistant *Pseudomonas aeruginosa* isolates from South Korea. Int. J. Antimicrob. Agents.

[33] Zeng ZR (2014). Mechanisms of carbapenem resistance in cephalosporin-susceptible *Pseudomonas aeruginosa* in China. Diagn. Microbiol. Infect. Dis..

[34] Favre-Bonté S, Köhler T, Van Delden C (2003). Biofilm formation by *Pseudomonas aeruginosa*: role of the C4-HSL cell-to-cell signal and inhibition by azithromycin. J. Antimicrob. Chemother..

[35] Alav I, Sutton JM, Rahman KM (2018). Role of bacterial efflux pumps in biofilm formation. J. Antimicrob. Chemother..

[36] Westfall LW (2006). *mvaT* mutation modifies the expression of the *Pseudomonas aeruginosa* multidrug efflux operon *mexEF-oprN*. FEMS Microbiol Lett..

[37] Strateva T, Mitov I (2011). Contribution of an arsenal of virulence factors to pathogenesis of *Pseudomonas aeruginosa* infections. Ann. Microbiol..

[38] Köhler T, van Delden C, Curty LK, Hamzehpour MM, Pechere JC (2001). Overexpression of the MexEF-OprN multidrug efflux system affects cell-to-cell signaling in *Pseudomonas aeruginosa*. J. Bacteriol..

[39] Clinical Laboratory Standards Institute. Performance standards for antimicrobial susceptibility testing. Twenty-seventh informational supplement M100-S27. CLSI, Wayne. (2017).

[40] Palma N (2017). Resistance to quinolones, cephalosporins and macrolides in *Escherichia coli* causing bacteraemia in Peruvian children. J. Global Antimicrob. Resist..

[41] Rodríguez MC (2010). Molecular characterization of *Pseudomonas aeruginosa* isolates in Cantabria, Spain, producing VIM-2 metallo-beta-lactamase. Enferm. Infecc. Microbiol. Clin..

[42] Chalhoub H (2016). High-level resistance to meropenem in clinical isolates of *Pseudomonas aeruginosa* in the absence of carbapenemases: role of active efflux and porin alterations. Int J Antimicrob Agents..

